# Comparison of ultrasound probe location and sonographic findings used for the evaluation of pneumothorax in canine cadavers: a pilot study

**DOI:** 10.3389/fvets.2026.1707807

**Published:** 2026-05-25

**Authors:** Ashley Finch, Søren R. Boysen, Valerie Madden, Phillipa J. Johnson, Madison Hillstead, Cole Harding, Katrine Gillett, Julie Menard

**Affiliations:** 1 Faculty of Veterinary Medicine, University of Calgary, Calgary, AB, Canada; 2 Paramount 24 Hour Animal Hospital, Calgary, AB, Canada; 3 VEG Emergency for Pet, White Plains, NY, United States; 4 College of Veterinary Medicine, Cornell University, Ithaca, NY, United States; 5 Faraday Veterinary Neuroimaging, Totnes, United Kingdom

**Keywords:** chest tube site, curtain sign, lung point, lung sliding, PLUS, POCUS

## Abstract

**Introduction:**

This pilot study aimed to compare sonographic findings at thoracic sites used to detect pneumothorax in canine cadavers.

**Methods:**

Intubated frozen–thawed cadavers without pre-existing sonographic evidence of pneumothorax were included. Control, unilateral and bilateral pneumothorax groups were created, with the latter induced by infusion of air (3 mL/kg) under ultrasound guidance. Four blinded sonographers (two experts and two novices) evaluated positive-pressure-ventilated (PPV) cadavers placed in sternal recumbency. Lung sliding and B-lines were assessed at the chest tube site (CTS) and caudo-dorsal border (CDB), while the abnormal abdominal curtain sign (AACS) was evaluated along the abdominal curtain sign (ACS). When absence of lung sliding was noted, operators searched for a lung-point (LP). Presence or absence of pneumothorax was recorded for the CTS, CDB, AACS, combined CTS + LP, and CDB + AACS + LP (Modified PLUS). Post-study right and left horizontal beam radiography was used as the reference standard to quantify pneumothorax volume by a board-certified radiologist. Results were analyzed by Fisher’s exact test with a statistical significance set at *p* < 0.05.

**Results:**

Mild pneumothorax was present in 10/16 hemithoraces, scant pneumothorax in 3/16, and no pneumothorax in 3/16. Combined accuracy, sensitivity, and specificity of all operators was 22% (9–40), 4% (0–20), 100% (54–100) for both CTS and CTS + LP; 53% (35–71), 42% (23–63), 100% (54–100) for CDB; 31% (16–50), 15% (4–35), 100% (54–100) for AACS; and 56% (38–74), 46% (27–67), 100%(54–100) for Modified PLUS, respectively. There was a significant difference in identification of pneumothorax between the CTS and CDB (*p* = 0.00027), and CTS and Modified PLUS (*p* = 0.0012) and between CTS + LP and Modified PLUS for all operator comparisons (*p* = 0.00012).

**Discussion:**

The site assessed (CDB vs. CTS) for lung sliding and the sonographic signs (AACS, lung sliding) evaluated with different POCUS protocols can influence the accuracy of diagnosing pneumothorax in PPV canine cadavers placed in sternal recumbency.

## 1 Introduction

Pneumothorax is a frequent cause of respiratory distress in small animals, often resulting from trauma or spontaneous lung rupture. Rapid identification is critical to enable thoracocentesis when clinically indicated, which relieves positive intrapleural pressure. While lung ultrasound is a reliable and accurate diagnostic tool in humans for identifying pneumothorax, its reliability in veterinary medicine remains less established, due to a lack of standardized methodology, poorly defined anatomical boundaries for free pleural air localization, and the sonographer’s level of expertise (1–6).

Point of care ultrasound (POCUS) leverages specific sonographic signs at various anatomical sites to either rule out or confirm pneumothorax. Key “rule-out” signs (in the absence of a lung point) at a specific probe location include the presence of B-lines, tissue-like patterns, normal lung sliding, lung pulse, and a normal abdominal curtain sign (ACS). Conversely, pneumothorax should be suspected when there is an absence of the above listed findings, and the diagnosis is further supported when an abnormal abdominal curtain sign (AACS) and/or a lung point (LP) is identified (4–6).

Unlike human medicine, evidence-based recommendations and the accuracy of lung ultrasound for diagnosing pneumothorax in veterinary medicine remains limited, with available data derived from only a limited number of published studies involving small patient populations (1–6). Most published veterinary studies rely solely on the absence of lung sliding for pneumothorax diagnosis, but the true sensitivity (Se) and specificity (Sp) of lung sliding to diagnose pneumothorax is uncertain due to the lack of comparison with accepted reference standards and/or the inclusion of only small case numbers (1–6). A clinical case series in dogs identified AACSs and LPs, in addition to the absence of lung sliding in cases with confirmed pneumothorax based on aspiration of pleural air from a chest tube, radiographs and/or computed tomography (CT). However, this study did not compare all cases with CT and included only six dogs (5). A controlled experimental study inducing small-volume pneumothorax (2–10 mL/kg of air) in anesthetized dogs (*n* = 9) described the novel reverse sliding sign along with lung sliding, lung pulse, LP, and the barcode sign when compared with CT (4). The study showed that lung sliding for detection of pneumothorax was 11, 56, 67%, for volumes of 2, 5, and 10 mL/kg, respectively, with a Sp of 89% regardless of the volume of air infused. It also showed that the LP and reverse sliding sign was more sensitive than lung sliding for the diagnosis of smaller volume pneumothorax. However, the findings of the Hwang et al. study have not been validated in clinical cases of naturally occurring or larger-volume pneumothorax.

Despite the growing use of pleural and lung ultrasound to support a diagnosis of pneumothorax in veterinary medicine, significant gaps remain in the research supporting its optimal application. Notably, no studies have directly compared different thoracic sites to determine the most sensitive locations for pneumothorax detection in small animals, leaving uncertainty regarding the best scanning protocol to use. For example, early thoracic focused assessment with sonography for trauma (FAST) protocols scanned patients in lateral recumbency using the chest tube site (CTS) to look for pneumothorax (3), although validation of this site for localization of free pleura air through comparison with reference standards is lacking. Furthermore, current recommendations are to scan veterinary patients with respiratory distress in a standing or sternal position, or the position they are least stressed (5, 7–9), however the CTS is still described as the site to look for pneumothorax despite changing the patient’s position. Others have suggested using sonographically defined borders to locate the most caudo-dorsal site and to look for free pleural air in sternal or standing patients (5), although the Se of different sites of the thorax of small animals have not been compared or validated. Additionally, while the AACS has been described as a potential indicator of pneumothorax, its diagnostic performance has not been systematically compared to other sonographic findings, or reference standard methods, limiting its clinical utility. Research on the accuracy of the LP in veterinary patients is also limited, with only a few case studies evaluating its utility in naturally occurring cases (5).

The purpose of this exploratory pilot study was to compare different sonographic findings and thoracic sites to identify small volume pneumothorax in canine cadavers placed in sternal recumbency. More specifically to compare a modified Calgary pleural and lung ultrasound (PLUS) (8) approach to locate the most caudal dorsal site +/− LPs and AACSs to the CTS ± LP to detect pneumothorax, as assessed by both expert and novice sonographers. Given the pilot nature of this investigation, results are considered exploratory. Should clinically relevant differences between techniques or scanning sites be identified, these findings will be used to inform power analysis and sample size calculations for future prospective clinical trials in live dogs, using CT as the reference standard. We hypothesized that there would be differences in the Se and Sp of POCUS findings between probe locations and characteristics assessed, and that operator experience would influence the detection of pneumothorax.

## 2 Materials and methods

### 2.1 Study design

This was a randomized cadaver study. The study was conducted at the University of Calgary, Faculty of Veterinary Medicine (Alberta, Canada). Ethical approval for the study protocol was granted by the University of Calgary Animal Care Council (AC23-0021). No animal was euthanized for the purpose of this study.

### 2.2 Cadaver selection and preparation

Thirteen thawed frozen canine cadavers were used. All fur was clipped over each hemithorax from the thoracic inlet to the last rib, using a #40 clipper blade. All sonographic scans were performed with cadavers positioned in sternal recumbency. Cadavers were intubated, tracheal cuffs were inflated, and positive-pressure-ventilation (PPV) was administered via an Ambu-bag (Ambu^®^ Resuscitators, Ambu, Denmark). Cadavers were included in the study when the absence of pneumothorax was confirmed based on presence of lung sliding and/or B-lines at the most caudodorsal border (CDB) bilaterally and a normal ACS was identified along the entire lateral-caudal lung margin with the probe placed perpendicular to the ribs, and the marker directed cranially. Imaging depth was generally set at 5–8 cm depending on the size of the cadaver, with the focus position set at or just beyond the pleural line. Alcohol was applied as the contact medium. To assess lung sliding the depth was decreased to the lowest setting that still allowed visualization of the pleural line, the gain was decreased, motion filters turned off, and the probe was fanned and/or situated over a rib to optimize the ability to identify lung sliding. The machine settings were then reset to the initial settings listed above for the remainder of the cadaver scanning. Cadavers were excluded as controls if there was an absence of lung sliding and an absence of B-lines noted at the CDB, the presence of a LP, or an AACS was identified by an expert sonographer (> 15 years of small animal lung ultrasound scanning experience) using a Sonosite Edge II (Fujifilm SonoSite inc. USA) ultrasound machine with a curvilinear probe (C11x/8–5 mHz transducer). In addition, dogs were excluded from the pneumothorax group if moderate pneumothorax was identified at baseline. If scant pneumothorax was identified these dogs were included in the pneumothorax group, and a decision was made to add air (up to 3 mL/kg) based on the initial baseline estimate of the pneumothorax. The preliminary sonographic scan performed by the unblinded expert sonographer was designated as baseline pathology and was recorded (see Supplemental Table 1). All cadavers were anticipated to have varying levels of pleural effusion secondary to freeze thaw artifact.

The order of hemithoraces assigned to either no pneumothorax or pneumothorax was randomized using an online-based randomization program. Research Randomizer (Version 4.0) [Computer software]. Pneumothorax was created in 8 hemithoraces by infusion of up to 3 mL of room air (depending on the pre-existing presence of small volume pneumothorax) into a small pocket of pleural effusion that was identified in all frozen thawed cadavers in the most gravity dependent, cranial ventral region of the pleural space. With the probe parallel to the ribs, under ultrasound guidance, a 14G catheter (14G, 2” Terumo Surflo, Terumo Medical Canada Inc. Ontario) connected to an extension set (Baxter IV extension set Clearlink ™ 16 Inch tubing, Mississauga, Ontario), 3-way stop cock (Baxter, Mississauga, Ontario), and a 30 cc syringe (Baxter Mississauga, Ontario), was guided into the small pocket of pleural effusion and 3 mL/kg of room air was infused. The quantity of air to be infused into the pleural space was determined based on an unpublished pilot study by the authors where canine cadavers positioned in sternal recumbency were assessed for the degree of pneumothorax using CT following infusions of 1, 3, 5 or 10 mL/kg of air. Pneumothorax was consistently identified by novice and expert sonographers following infusion of 3 mL/kg of air in the pilot study.

### 2.3 Data collection

Two experts (DACVECC specialists with more than 5 years of POCUS experience) and 2 novices (4th year veterinary students with minimal POCUS experience) participated in a blinded cross over study. Novice sonographers underwent 1 h of training to familiarize themselves with each protocol. One novice and one expert were assigned to perform either the CTS + LP or modified PLUS protocol on each hemithorax. The other novice and expert were assigned to the opposite protocol. As such, each hemithorax was scanned by an expert and novice using both protocols using an S9 Sonoscape ultrasound machine (Sonoscape Medical Corp., Guangzhou, China) with a medium-frequency (4–8 MHz) curvilinear probe. Each sonographer recorded their findings and the length of time to complete the POCUS examination. The initial group’s protocol was randomly assigned using an online-based randomization program (10).

**Figure 1:**
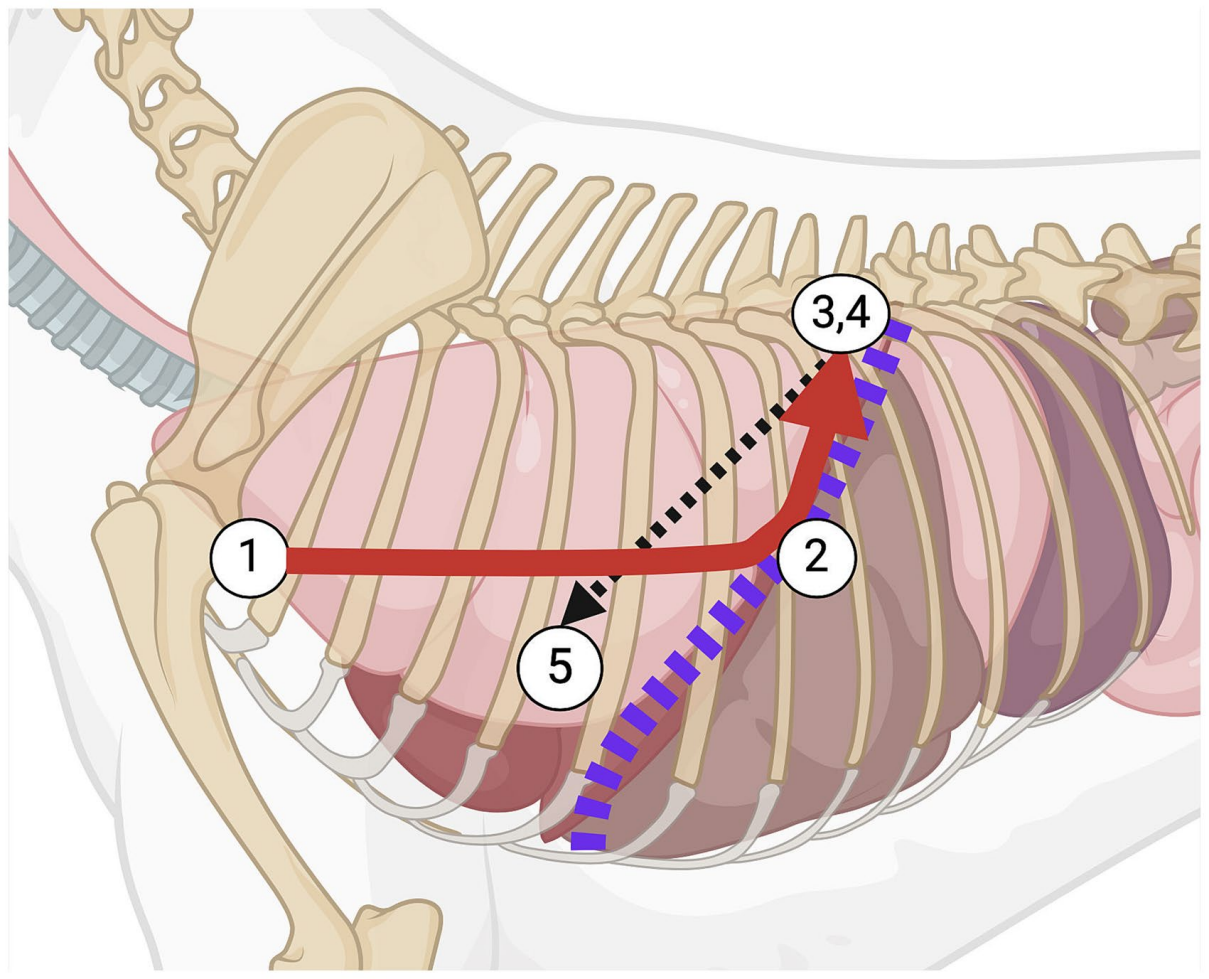
Modified PLUS. (1) Starting point: the probe was placed perpendicular to the ribs with the probe marker directed cranially, ½ way up the thorax just caudal to the triceps muscles, and the presence or absence of lung sliding and/or B-lines was assessed. The probe was then slid caudally until (2) the abdominal curtain sign (ACS) was identified. The ACS was assessed as being normal or abnormal. The ACS was then followed dorsally until the iliocostalis muscles were identified (3). The probe was then slowly swept ventrally off the iliocostalis muscles until the pleural line reappeared. (4) This was considered the caudo-dorsal border (CDB). At this site, the ACS was evaluated as being normal or abnormal and the pleural line was assessed for the presence/absence of lung sliding and the presence/absence of B-lines. If a pneumothorax was suspected (based on an AACS and/or absence of lung sliding), the lung point (LP) was sought by sliding the probe towards the elbow.

**Figure 2:**
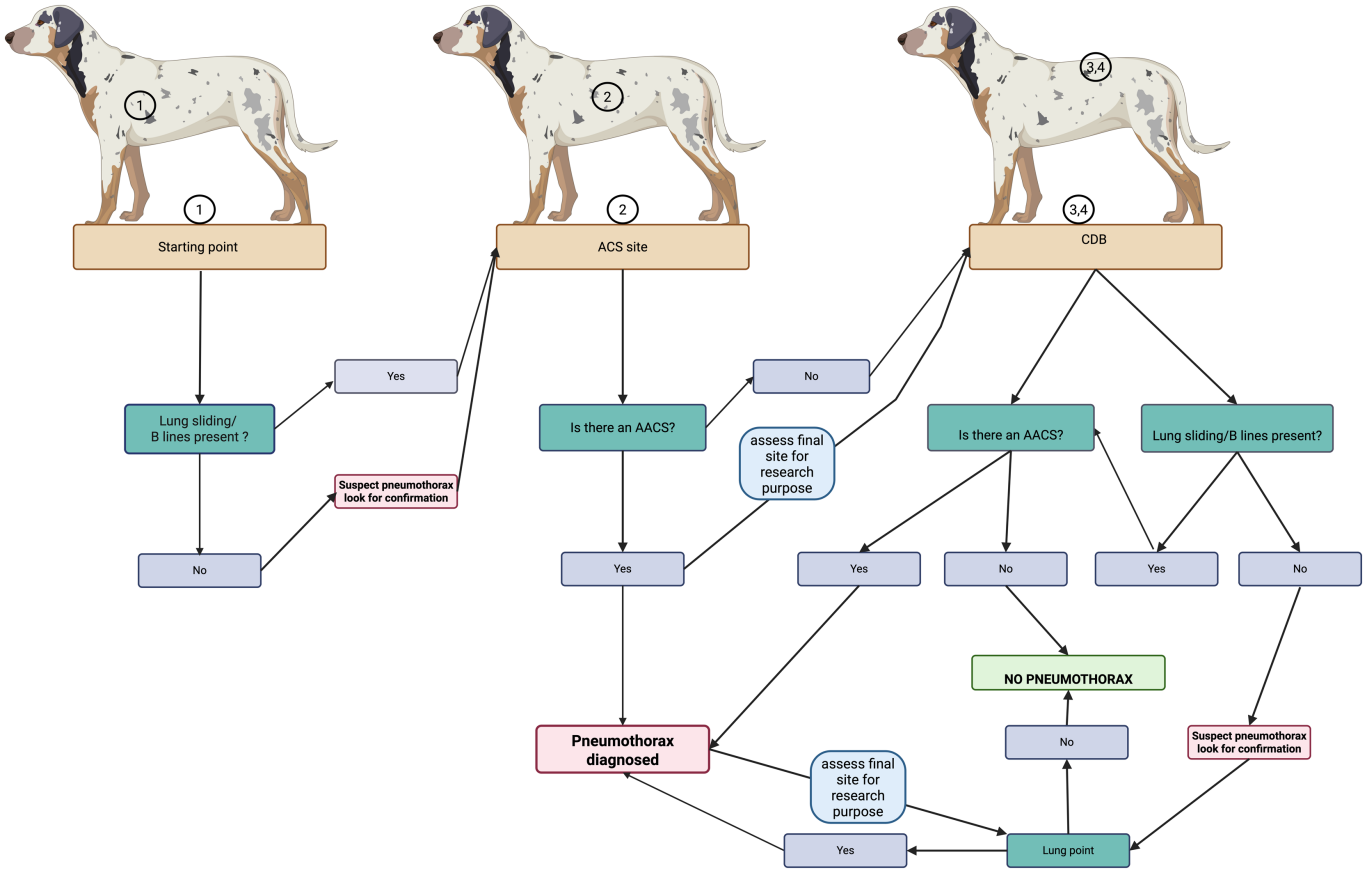
Research driven flow chart for modified PLUS. Nota bene: In live patients, identification of an abnormal abdominal curtain sign (AACS) would be diagnostic of pneumothorax. Absence of a lung point could be due to a massive pneumothorax. In this study, the pneumothorax volume was controlled, hence this specific flow chart was used to make a diagnosis regarding the presence/absence of pneumothorax. ACS, abdominal curtain sign; CDB, caudal dorsal border.

The Calgary PLUS protocol (Figure 1) was modified (Modified PLUS) to focus on pneumothorax findings and did not include a search for pleural effusion, and did not scan the more ventral lung surfaces for pathology. It began with the probe being placed perpendicular to the ribs midway between the sternum and spine, just caudal to the muscles of the front limb (see Figure 1). At this starting point the presence or absence of lung sliding and/or B-lines were assessed. The probe was then slid caudally until the abdominal curtain sign (ACS) was identified. The ACS was assessed as normal or abnormal (AACS), with a normal ACS defined as a sharply demarcated vertical edge separating aerated lung from abdominal contents, and the vertical edge artifact moving in synch with the abdominal contents during the respiratory phases of PPV. The presence of a normal ACS made pneumothorax less likely at the probe location. The ACS was then followed dorsally, until the iliocostalis muscles were identified, ensuring AACSs did not appear. The probe was then slowly swept ventrally off the iliocostalis muscles until the pleural line reappeared. This was considered the sonographically defined CDB. At the CDB, under PPV (provided by an assistant), presence or absence of lung sliding and/or B-lines was assessed. The presence of lung sliding and/or B-lines ruled out pneumothorax at this probe location. If lung sliding and B-lines were absent, pneumothorax was suspected, and the probe was slid from the area of absent lung sliding towards the lung hilus (towards the elbow) to search for a LP. The lack of lung sliding and/or B-lines at the CDB and/or identification of an AACS and/or LP were considered positive for presence of pneumothorax (Figure 2). All sites described and assessed above, which included the starting point, the ACS, the CDB and combined CDB+ ACS+LP (Modified PLUS) were recorded.

**Figure 3:**
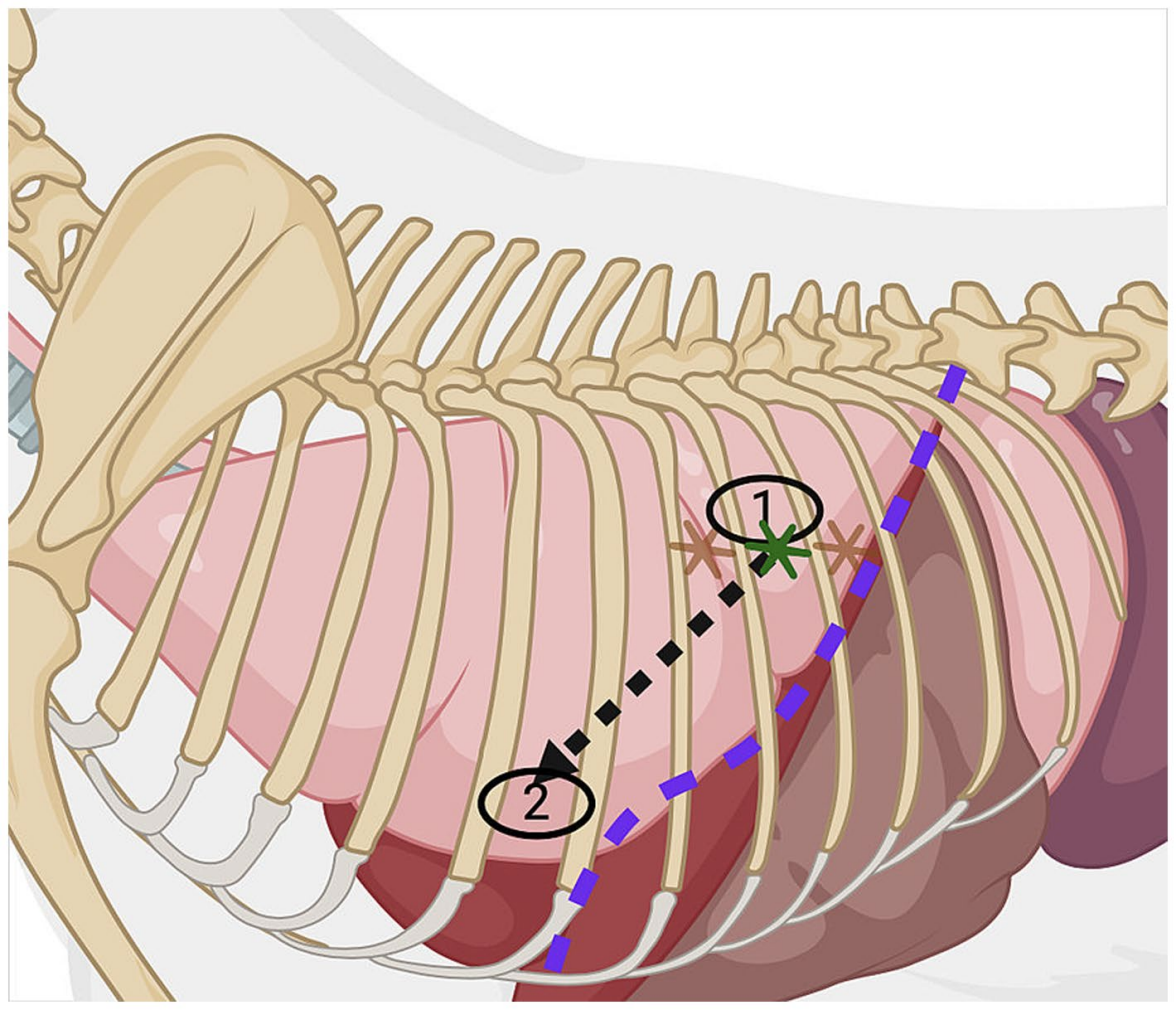
Modified chest tube site (CTS) and lung point (LP) scanning protocol. (1) For the CTS protocol, the sonographer identified the subxiphoid site and placed the probe perpendicular to the ribs, with the marker directed cranially in the upper one third of the thorax (9). If the curtain sign was visualized, the probe was then slid forward 2–3 intercostal spaces to the CTS site (approximately the 7th (amber star), 8th (green star), or 9th (amber star) intercostal space). The CTS was assessed for absence of lung sliding and/or presence of B-lines under positive pressure ventilation (PPV). (2) If lung sliding was absent, a LP was sought by sliding the probe towards the elbow (black arrow).

**Figure 4:**
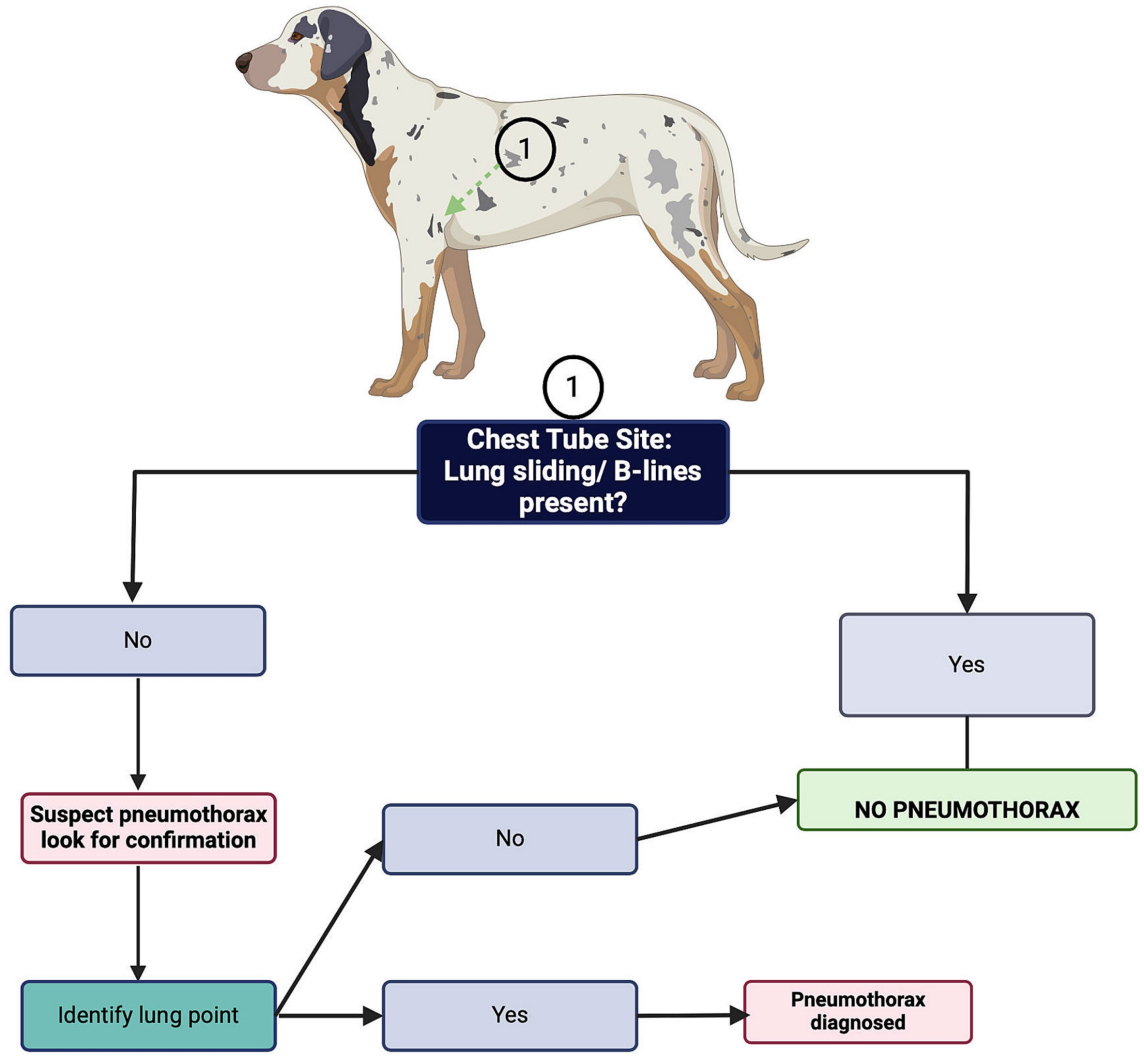
Research driven flow chart for the chest tube site (CTS). In live patients, absence of a lung point (LP) could be due to a massive pneumothorax. In this study, the volume of pneumothorax was controlled, hence this specific flow chart was used to make a diagnosis on presence/absence of pneumothorax.

For the CTS + LP protocol (Figure 3), the sonographer identified the subxiphoid site and placed the probe perpendicular to the ribs at the upper one third of the thorax (9). If the curtain sign was visualized, the probe was then slid forward 2–3 intercostal spaces to the CTS site (approximately the 7th-9th intercostal space). The CTS was assessed for presence or absence of lung sliding and/or B-lines under PPV. If lung sliding was absent the probe was slid from the area of absent lung sliding towards the lung hilus (towards the elbow) to search for the LP. If lung sliding and/or B-lines were present, pneumothorax was ruled out (Figure 4). Positive or negative pneumothorax findings at the CTS and combined CTS + LP were recorded.

### 2.4 Reference standard

**Figure 5:**
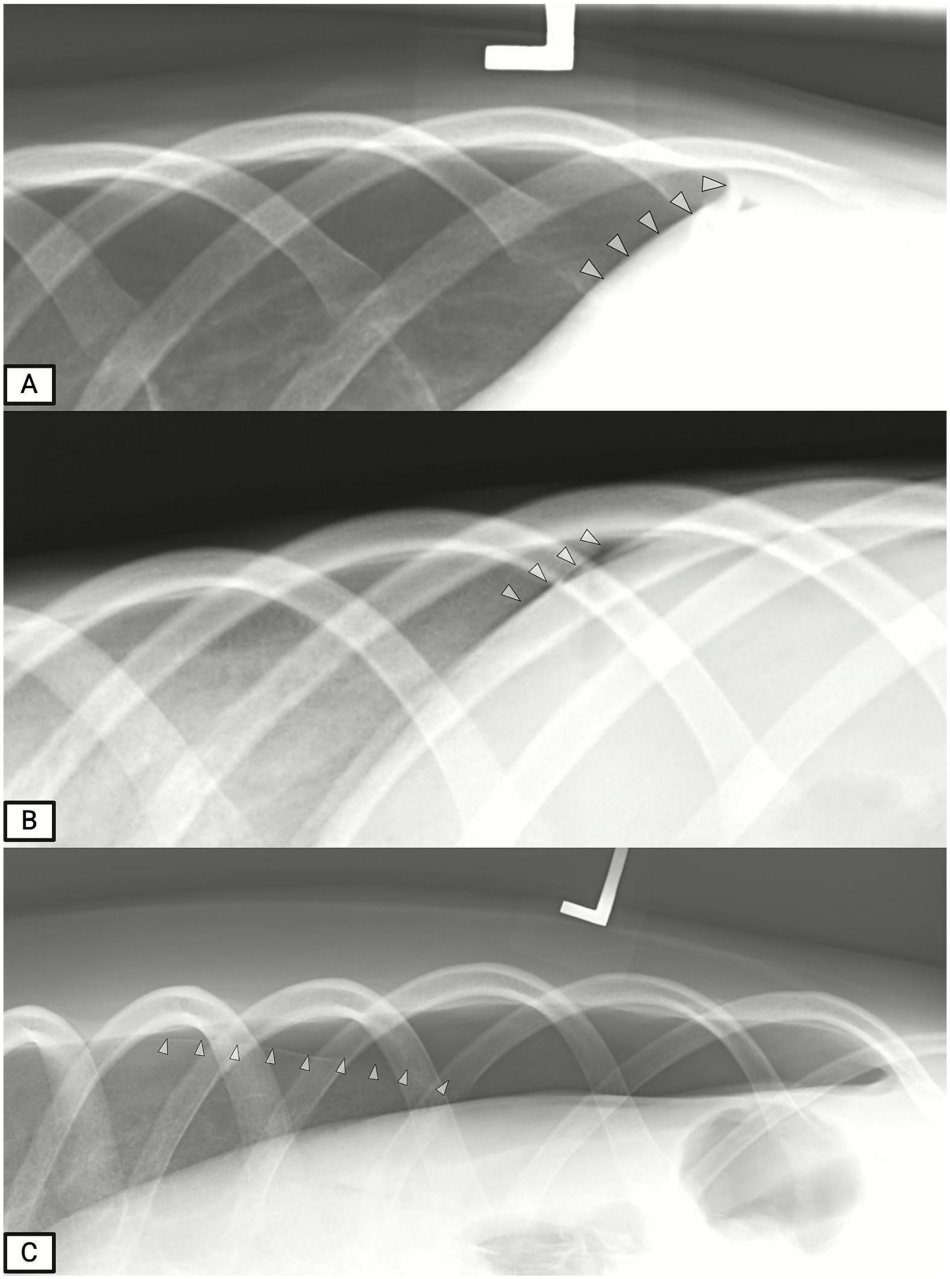
Horizontal plane ventral to dorsal projections of the left caudal hemithorax in three cadavers. The white arrow heads demonstrate the visceral pleural margin of the left caudal lung lobe. **(A)** Absent pneumothorax. **(B)** Scant pneumothorax. **(C)** Mild pneumothorax.

Within 6 h of completion of the study, all cadavers underwent post-study POCUS examination by the same independent experienced POCUS sonographer that performed the initial assessment of cadavers. This was done to ensure that no new pathology was introduced or developed during data collection. The presence or absence of a pneumothorax and location of the LP in cases with pneumothorax was recorded. Following POCUS data collection, reference standard evaluation of the presence or absence of pneumothorax was assessed via thoracic radiographs (Innovet DXR^™^, CXDI Control software NE, Canon Inc. 2012). The same day cadavers were scanned, each cadaver had post-study 2-view thoracic radiography (right lateral and left lateral horizontal beam) performed. Horizontal beam radiography was used as the reference standard for detection of free pleural air (11). A blinded board-certified radiologist reviewed the DICOM images and classified the absence or presence of pneumothorax with a modified grading scale from Lynch et al. (11), with scant, mild, moderate, and severe pneumothorax (Figure 5.)

### 2.5 Statistical analysis

A sample size calculation was performed to compare the Se and Sp of the TFAST as reported by Walters et al. (1) with a Se of 54% and Sp of 81%, and Hwang et al. (4) with the reverse lung sliding using a 2 mL/kg volume pneumothorax with Se of 89% and Sp of 100%. Assuming a disease prevalence of 50%, a significance level of 0.05, and 80% power, a minimum of 8 diseased and 8 non-diseased hemithoraces (total *n* = 16 hemithoraces, 8 dogs) was required to detect a statistically significant difference.

Normalcy was determined via the D’Agostino and Pearson test. Descriptive statistics were calculated. The difference between time to scan between novices and experts and scanning protocols were compared with a paired t test. For each individual site, sonographer experience, accuracy, Se, and Sp were determined. Additionally, the combined accuracy, Se, and Sp of all operators was also determined for each point and the combined CTS + LP and Modified PLUS protocols. Categorical results were analyzed by Fisher’s exact or Chi square test using a commercial software GraphPad Prism version 10.0 (GraphPad Software, Inc., San Diego, CA). For statistical analysis, results were calculated two ways: one classifying scant air as positive for pneumothorax and the second considering it negative, given it would unlikely be detectable on conventional orthogonal radiographs and POCUS. A *p* value of ≤ 0.05 was considered significant for all comparisons.

## 3 Results

Thirteen canine cadavers were available for the research (Supplementary file 1). Five cadavers were eliminated because pneumothorax could not be ruled out with the above-mentioned criteria. Eight medium sized canine cadavers of unknown age and various breeds were included in the study (mean weight: 22.3 kg (range 30.1–13.5 kg); six females and two males). According to the radiologist’s assessment of post study horizontal beam radiographs, pneumothorax was present in 13 out of 16 hemithoraces, with 3/12 classified as scant and 10/16 as mild.

### 3.1 Time to scan

Novice sonographers, but not experts, took significantly longer to perform Modified PLUS compared to the CTS + LP (Table 1). Overall, with experts and novices combined, CTS + LP was significantly faster to complete than Modified PLUS.

**Table 1:** Protocol scan times in minutes for novice, expert and combined expert and novice sonographers.

Time in minutes	Modified PLUS	CTS + LP	*p* value
Novice	15.2 Std: 5.9	7.1 STD: 1.3	**0.0068**
Expert	5.2 STD: 1.8	3.6 STD: 1.2	0.1129
Combined time (expert + novice)	10.2 STD: 3.8	5.4 STD: 1.0	**0.0138**

Values are mean and standard deviation. Bold *p*-values indicate statistically significant differences. PLUS, pleural space and lung ultrasound; CTS + LP, chest tube site + lung point.

### 3.2 Probe site and diagnostic accuracy

The search for a LP after identifying the absence of lung sliding at the CTS did not change the accuracy, Se or Sp of the CTS + LP protocol. (Tables 2, 3). The CTS + LP protocol had a negative predictive value (NPV) of 19 to 31%, suggesting it can miss cases when a pneumothorax is present.

**Table 2:** Summary of sensitivity, specificity, accuracy, negative predictive value (NPV) and positive predictive value (PPV) of the CTS and CTS + LP for identification of pneumothorax when compared to the reference standard of horizontal beam radiography.

Site assessed	Sonographer	Specificity (%) (95% CI)	Sensitivity (%) (95% CI)	Accuracy (%) (95% CI)	PPV (%) (95% CI)	NPV (%) (95% CI)
Lung sliding CTS, LP and overall protocol (Lung sliding CTS + LP)	Novice	100 (29–100)	0 (0–25)	19 (4–46)	0	19 (19–19)
	Expert	100 (29–100)	7 (0–36)	25 (7–52)	100 (2.5–100)	20 (18–23)
	All	100 (54–100)	4 (0–20)	22 (9–40)	100 (2.5–100)	19 (18–21)

CTS, chest tube site; LP, lung point.

**Table 3:** Summary of sensitivity, specificity, accuracy, negative predictive value (NPV) and positive predictive value (PPV) of the CTS and CTS + LP for identification of pneumothorax when compared to the reference standard of horizontal beam radiography.

Site assessed	Sonographer	Specificity (%) (95% CI)	Sensitivity (%) (95% CI)	Accuracy (%) (95% CI)	PPV (%) (95% CI)	NPV (%) (95% CI)
Lung sliding CTS, LP and overall protocol (lung sliding CTS + LP)	Novice	100 (54–100)	0 (0–31)	37.5 (15–65)	0	37.5 (15–65)
	Expert	83 (36–100)	0 (0–31)	31 (26–42)	0	33 (26–42)
	All	92 (62–100)	0 (0–17)	34 (19–53)	0	35 (32–39)

CTS, chest tube site; LP, lung point. Scant pneumothorax was considered a negative pneumothorax, as it would not be detected on traditional orthogonal radiographic view and is not considered clinically significant.

For the Modified PLUS protocol, identification of the absence of lung sliding at the starting point was the least sensitive location to diagnose a pneumothorax, whereas identification of the LP had the highest Sp, Se, accuracy and NPV. Experts had the highest accuracy for identification of an AACS compared to novices (Tables 4, 5). When combining the experts’ and novices’ findings, there was a significant difference in correct identification of pneumothorax between the CTS + LP and CDB (*p* = 0.0027), CTS and Modified PLUS (*p* = 0.0012), CTS and LP (*p* = 0.0012) but not for CTS and AACS (*p* = 0.3547). There was no difference in the number of true positive/ false negative for identification of AACS between novices and experts (*p* = 0.0957).

**Table 4:** Summary of sensitivity, specificity, accuracy, negative predictive value (NPV) and positive predictive value (PPV) of lung sliding sites and characteristics assessed via Modified PLUS for identification of pneumothorax when compared to the reference standard of horizontal beam radiography.

Site assessed	Sonographer	Specificity (%) (95% CI)	Sensitivity (%) (95% CI)	Accuracy (%) (95% CI)	PPV (%) (95% CI)	NPV (%) (95% CI)
Lung sliding behind elbow	Novice	100 (29–100)	0 (0–25)	19 (4–46)	–	19 (19–19)
	Expert	100 (29–100)	15 (2–45)	31 (11–59)	100(16–100)	21 (18–26)
	All	100 (54–100)	8 (1–25)	25 (11–43)	100 (16–100)	20 (18–22)
Abnormal asynchronous curtain sign (AACS)	Novice	0 (0–25)	100 (29–100)	19 (4–46)	–	19 (19–19)
	Expert	100 (29–100)	31 (9–61)	44 (20–70)	100 (40–100)	25 (19–32)
	All	100 (54–100)	15 (4–35)	31 (16–50)	100 (40–100)	21 (19–24)
Caudo-dorsal border (CDB)	Novice	100 (29–100)	31 (9–61)	44 (20–70)	100 (40–100)	25 (19–32)
	Expert	100 (29–100)	54 (25–81)	62.5 (35–84)	100 (59–100)	33 (22–47)
	All	100 (54–100)	42 (23–63)	53 (35–71)	100 (72–100)	29 (22–36)
Lung point (LP)	Novice	100 (29–100)	39(14–68)	50 (25–75)	100 (48–100)	28 (20–37)
	Expert	100 (29–100)	54 (25–81)	62.5 (35–84)	100 (59–100)	33 (22–47)
	All	100 (54–100)	46 (27–67)	56 (38–74)	100 (74–100)	30 (23–38)
Modified PLUS (lung sliding, AACS, CDB, LP)	Novice	100 (29–100)	39(14–68)	50 (25–75)	100 (48–100)	28 (20–37)
	Expert	100 (29–100)	54 (25–81)	62.5 (35–84)	100 (59–100)	33 (22–47)
	All	100 (54–100)	46 (27–67)	56 (38–74)	100 (74–100)	30 (23–38)

AACS, abnormal asynchronous curtain sign; CDB, Caudo-dorsal border; LP, lung point; PLUS, pleural space and lung ultrasound.

**Table 5:** Summary of sensitivity, specificity, accuracy, negative predictive value (NPV) and positive predictive value (PPV) of lung sliding sites and characteristics assessed via Modified PLUS for identification of pneumothorax when scant pneumothorax detected by horizontal beam radiography was determined to be absent.

Site assessed	Sonographer	Specificity (%) (95% CI)	Sensitivity (%) (95% CI)	Accuracy (%) (95% CI)	PPV (%) (95% CI)	NPV (%) (95% CI)
Lung sliding behind elbow	Novice	100 (54–100)	0 (0–31)	37.5 (15–65)	---	37.5 (37.5)
	Expert	100 (54–100)	20 (3–56)	50 (25–75)	100(16–100)	43 (36–51)
	All	100 (74–100)	10 (1–32)	44 (26–62)	100 (16–100)	40 (37–44)
Abnormal asynchronous curtain sign (AACS)	Novice	100 (54–100)	0 (0–31)	37.5 (15–65)	---	37.5 (37.5)
	Expert	100 (59–100)	44 (14–79)	69 (41–89)	100 (40–100)	58 (41–88)
	All	100 (75–100)	21 (6–46)	53 (35–71)	100 (40–100)	46 (41–52)
Caudo-dorsal border (CDB)	Novice	100 (29–100)	40 (12–74)	62.5 (35–84)	100 (40–100)	50 (38–62)
	Expert	100 (54–100)	70 (35–93)	81 (54–96)	100 (59–100)	67 (44–88)
	All	100 (74–100)	55(32–77)	72 (53–86)	100 (72–100)	57 (53–86)
Lung point (LP)	Novice	100 (54–100)	50(19–81)	69 (42–89)	100 (48–100)	55 (39–69)
	Expert	100 (54–100)	60 (26–88)	75 (48–93)	100 (54–100)	60 (41–76)
	All	100 (74–100)	55 (32–77)	72 (53–86)	100 (72–100)	57 (45–68)
Modified PLUS (lung sliding, AACS, CDB, LP)	Novice	100 (54–100)	50(19–81)	69 (42–89)	100 (48–100)	55 (39–69)
	Expert	100 (54–100)	70 (35–93)	81 (54–96)	100 (59–100)	67 (44–88)
	All	100 (74–100)	60 (36–80)	75 (57–89)	100 (74–100)	60 (47–72)

AACS, abnormal asynchronous curtain sign; CDB, Caudo-dorsal border; LP, lung point; PLUS, pleural space and lung ultrasound.

## 4 Discussion

In this study using canine cadavers undergoing PPV in sternal recumbency with small-volume pneumothorax, the accuracy of pneumothorax detection via POCUS varied depending on the diagnostic criteria used and the thoracic site assessed. Diagnosis was most accurate when a LP was identified, when lung sliding was absent at the CDB, or when all sonographic characteristics of pneumothorax were assessed together using the Modified PLUS protocol, compared to assessing a single site such as the CTS. However, when using the combination of absent lung sliding at the CDB and an AACS alone, the overall accuracy for pneumothorax diagnosis was only 56%.

The initial study design included eight dogs with pneumothorax and eight control dogs. However, post-study horizontal beam radiographic analysis by a board-certified radiologist revealed that 13 out of 16 cadavers had pneumothorax. Three dogs originally assigned to the control group were found to have very scant pneumothorax, and two additional control dogs developed a mild pneumothorax by the end of the study. The development of pneumothorax in the control group may have been secondary to the condition of the cadavers, which had been frozen, thawed, and subjected to PPV via an Ambu-bag. The fragility of the lung tissue could have contributed to the formation of pneumothorax at any point during the examination.

Horizontal beam radiography was chosen for pneumothorax detection because it is widely available in clinical practice for rapid assessment and has higher Se compared to ventrodorsal radiographic views (11). Radiographs were obtained as soon as possible after sonographic data collection, without additional PPV, and without altering patient position except during radiographic acquisition. Depending on the scanning and imaging sequence, radiographs were taken within 2 to 6 h post-scan. This length of time may have allowed pneumothorax to develop if it was not previously present or resulted in a change in the size of the pneumothorax. Using a modified grading scale from Lynch et al., pneumothorax in three dogs were classified as scant (Figure 5) (11). Given the minimal volume of air detected, these cases were likely not clinically significant and would be unlikely to be detected via standard orthogonal radiographic views or POCUS, as the air was positioned beneath the iliocostalis muscles. As expected, when scant pneumothorax was classified as absent, the Se, Sp, accuracy, and NPV increased.

Most veterinary studies to date have focused on the Se and Sp of individual POCUS findings rather than specific sites or a combination of multiple POCUS findings and the respective sites to assess those findings for diagnosing pneumothorax. For instance, Hwang et al. evaluated the absence of lung sliding, lung pulse, and LP, as well as the presence of reverse lung sliding within a 4-quadrant protocol in dogs in sternal position but did not report which sites were more sensitive for the specific signs (4). The most sensitive sign was reverse lung sliding, which demonstrated the highest Se, Sp, and predictive values for diagnosing pneumothorax.

Existing POCUS protocols, developed in both human and veterinary medicine, are based on the principle that pleural air rises to the widest, non-dependent thoracic regions, while pleural fluid gravitates to the most dependent areas (3–5, 12) The distribution of intrapleural air is influenced by lung elasticity and gravity, with air accumulating in the caudo-dorsal thorax and fluid pooling ventrally (8). Consequently, protocols such as Calgary PLUS emphasizing sonographically identifiable borders at the caudo-dorsal thorax are likely to improve Se, Sp, and accuracy in identifying smaller volume pneumothorax (5, 8). In the current study, the modified PLUS protocol was more accurate at identifying pneumothorax when evaluating findings and sites, including lung sliding at the starting point behind the triceps, the CDB, the LP, and the identification of an AACS along the length of the ACS. Among these sites, the absence of lung sliding at the starting point, located behind the triceps, approximately halfway up the thorax, was the least sensitive for pneumothorax detection. This finding is not surprising, as pneumothorax typically compresses the lungs from the CDB ventrally towards the hilus of the lungs. Novice sonographers identified lung sliding in all 16 cases, regardless of whether lung sliding was actually present. This may indicate that they scanned too far ventrally, failing to visualize the true absence of lung sliding.

The identification of an AACS also demonstrated lower Se and accuracy compared to other components of the modified PLUS protocol, particularly among novice sonographers. An AACS is a relatively recent sonographic finding associated with pneumothorax in dogs (5). Significant discrepancies were noted between expert and novice sonographers in identifying an AACS. Novice sonographers had lower Se and accuracy for detecting an AACS, further supporting the notion of a steeper learning curve for this specific skill. Standardization of training protocols and consensus statements to identify pleural space and lung pathology in veterinary medicine are lacking. Although novices were provided 1 h of individual training by highly experienced operators (> 20 years of veterinary lung ultrasound), it is likely that the familiarity of protocols applied by experts in clinical practice explain some of the difference between novices and experts noted in this study. Further research assessing the optimal number of scans and hours of training required to become proficient in veterinary lung ultrasound is needed. Additionally, these differences may reflect the limitations of our small sample size, potentially leading to a Type II error. A larger study cohort may help clarify the diagnostic utility of sonographic findings such as the LP and AACSs when incorporated into standardized protocols. Furthermore, PPV may alter the appearance of AACSs compared to spontaneously breathing dogs, potentially complicating detection. Furthermore, AACSs may have been reported by the sonographer at a single location directly opposite the starting point, rather than identification along the diaphragm. Given the small pneumothorax volumes in this study, AACSs may not have been visible at that specific site and may have been present more caudodorsally but not identified. Future studies in spontaneously breathing dogs are needed to better correlate the transition from a normal ACS to an AACS, the identification of a LP, and pneumothorax volume. Additionally, if an AACS was detected, pneumothorax was suspected. In clinical patients, POCUS scanning may stop. In our study, the scanning protocol was completed for research purposes and identification of an AACS may have influenced the extent to which the CDB site was evaluated, introducing potential bias.

The CDB is the most gravity-independent site, and as air rises, it should theoretically be the most sensitive location for detecting pneumothorax in dogs. However, this was not the case in our study, as novice sonographers sometimes failed to identify the absence of lung sliding at this site. This difficulty may have resulted from suboptimal ultrasound machine settings, which hindered the visualization of the pleural line and lung sliding, thereby reducing the ability to accurately assess lung movement. Although not specifically recorded in the study, because novices were not specifically instructed to optimize machine settings, they did not change or adjust them within the study. By contrast, experienced sonographers adjusted depth, focal position, frequency, harmonics and gain at their discretion, which may have impacted findings. Identifying lung sliding, or its absence, may become even more challenging in cases of moderate to large pneumothorax. In dogs experiencing respiratory distress, increased respiratory rates and effort could introduce additional obstacles to accurate identification. Further studies in live, non-anesthetized dogs, particularly those with traumatic or spontaneous pneumothorax, are necessary to refine the diagnostic performance of lung sliding criteria in these clinical scenarios. It is challenging to determine whether identification of lung sliding was reported in previous studies. Indeed, in the Hwang study (4), what was described as “lung sliding” may have actually been the expansion and retraction of A-lines in the presence of pneumothorax, with the identification of “reverse sliding” (motion in the opposite direction) being used for pneumothorax diagnosis. In contrast, our study assessed lung surface movement to determine lung sliding, and what Hwang et al. described as the reverse sliding sign may correspond to an asynchronous abnormal curtain sign (4, 5). In the Hwang et al. study, the reverse sliding sign was the most specific and sensitive finding for diagnosing small pneumothorax. However, as pneumothorax volume increased, the Se and Sp of reverse sliding decreased, possibly due to the emergence of a double curtain sign or other sonographic artifacts. Additionally, when assessing the CDB site, detection of an AACS would prompt clinicians to suspect pneumothorax and seek confirmation by identifying a LP. As discussed earlier, novices demonstrated lower accuracy in diagnosing AACSs which may have also influenced the result at the CDB site. A LP is a dynamic sonographic finding that marks the interface where the visceral and parietal pleura recontact in patients with pneumothorax. Identifying a LP is a critical diagnostic step that can guide therapeutic decisions. If a pneumothorax is classified as moderate to large, with a LP located in the ventral cranial thoracic region, thoracocentesis should be performed emergently. Conversely, when the LP is identified in the most dorsal region of the thorax, indicating a small pneumothorax, POCUS can be used for continued patient monitoring. In our study, LP identification had the highest Se, accuracy, and NPV for pneumothorax detection. Identifying a LP in a cadaver may be easier than in a live dog, particularly in frozen–thawed specimens where the pleural line appears irregular, discontinuous, or grainy. The presence of B-lines may further aid LP identification in cadavers. In spontaneously breathing dogs, especially those with non-traumatic pneumothorax, LP detection may be more challenging due to a smoother, more regular pleural line and the absence of B-lines. Further studies are needed to validate these findings in clinical cases of traumatic and non-traumatic pneumothorax.

The CTS demonstrated lower Se, accuracy, and NPV, compared with any of the modified PLUS protocol sites, which was unexpected. Since the CTS is located more caudodorsally than the modified PLUS starting point behind the triceps, the absence of lung sliding detected at the Modified PLUS starting point should also have been detected at the CTS. However, this was not the case, and the reason for this remains unclear. It is noteworthy that the CTS (named after the site a chest tube is placed) is typically more ventral than the caudo-dorsal border (3, 5, 8). In dogs with small-volume pneumothorax positioned sternally, the pneumothorax may not extend to the CTS, allowing lung sliding to persist. Alternatively, if the LP was located at the CTS, it is possible it may have been confused for lung sliding and erroneously resulted in pneumothorax being ruled out. This may contribute to the relatively low NPV observed in the current study. In cases with larger volume pneumothorax, which might be more clinically relevant, the accuracy of the CTS is expected to be higher. Another possible explanation for the decreased diagnostic ability of the CTS is the variability in locating the CTS in various breeds and sizes of dogs. Probe positioning was likely variable despite using the xiphoid as a landmark to guide probe placement along the upper two-thirds of the thorax. If placed too ventrally, the probe may be positioned ventral to the pneumothorax, allowing lung sliding to be identified.

For expert sonographers, the absence of lung sliding at the CDB was the most accurate and sensitive indicator of pneumothorax. When combining expert and novice data, LP identification remained the most accurate and sensitive diagnostic feature. However, expert assessment of pneumothorax at the CDB yielded an NPV of 33%, meaning that 67% of dogs assessed as not having pneumothorax at this location may still have had one. When scant pneumothorax was classified as negative (not clinically relevant), the NPV increased to 60%, indicating that 40% of dogs classified as pneumothorax-negative at this site may still have had a pneumothorax. These results align with those of Hwang et al., where the absence of lung sliding had low Se, moderate to high Sp, and an NPV of 50%. However, the calculated Se and Sp in the present study require further validation, as confirming the true absence of pneumothorax in the control group was inherently challenging, and a surrogate reference standard (horizontal beam radiography) was used in place of CT. Accordingly, the diagnostic accuracy estimates reported here should be interpreted with caution. The findings of this study are therefore exploratory in nature and require confirmation through prospective clinical investigations in a larger population of live dogs, incorporating CT as the reference standard.

In human trauma patients, POCUS-based detection of pneumothorax, utilizing absent lung sliding and the barcode sign on M-mode, has shown a Se of 89%, Sp of 95%, and NPV of 95% (13). One notable difference between human and veterinary studies is the respiratory control of patients during evaluation. Human patients, even in respiratory distress, can take deep breaths or hold their breath, potentially improving ultrasound diagnostic accuracy compared to CT. By contrast, in our study, the sonographer relied on an assistant to provide breaths without controlling or standardizing the respiratory rate or pattern. Although differences in respiratory control alone do not fully account for the observed disparity in Se and NPV between human and veterinary studies, incorporating additional sonographic findings, such as the barcode sign, may enhance the diagnostic accuracy and NPV of POCUS for detecting pneumothorax in canine trauma patients. Further refinement of protocols and techniques is warranted to optimize diagnostic performance in veterinary applications.

The limitations of this study pertain mostly to a cadaver study and small sample size. As a cadaver study, there is an inherent inability to completely remove all pathology from the pleural space due to freezing and thaw artifacts. As such, there was potential for bias to exist because of a lack of true negative control hemithoraces. Although experts and novices were blinded to the number and the presence or absence of pneumothorax, obtaining 8 true negative hemithoraces was not possible due to the challenge of sourcing canine cadavers and the fact approval for only 13 total cadavers was granted based on ethics approval. A larger sample size with more true negative controls should be considered. It was similarly impossible to ensure that each hemithorax was completely isolated from the other and that pathology would not redistribute between the independent sides. The impact of this possibility was minimized by scanning each cadaver prior to and post data collection to ensure that the amount of pathology remained as similar as possible for each sonographer. Additionally, each cadaver model was placed under PPV which cannot duplicate spontaneous respiration in a living, breathing animal. Furthermore, PPV could have created or expanded the volume of pneumothorax between sonographers, although this is not likely significant as the expert scanner verified and compared findings at the start and end of the study, prior to moving cadavers to radiography. Additional avenues of study include investigating the difference in protocols for different volumes of pneumothorax as the subcategory analysis between groups for scant, mild and moderate pneumothorax in the current study was not performed due to small sample size within categories. Because necropsy was not performed following the experimental procedures, the presence of pleural adhesions could not be assessed. As this study evaluated only specific thoracic regions (the CDB and CTS sites), it is possible that undetected pleural adhesions may have restricted intrapleural gas movement. If present, such adhesions could have compartmentalized air outside the scanned regions, potentially resulting in missed pneumothorax detection. We also did not evaluate the value of other sonographic findings (e.g., M-mode). Clinically, decision making for diagnosis of pneumothorax relies on a combination of signs, including POCUS findings with the key “rule-out” signs (in the absence of a LP) at a specific probe location including the presence of B-lines, tissue-like patterns, normal lung sliding, lung pulse, a normal ACS and key “rule in findings”: absence of the above listed findings and an AACS and/or a LP (4, 5, 8). Use of a linear probe may have increased detection of pleural line abnormalities and changed the results of our study, however further studies on the use of linear compared to microconvex probes for detection of pneumothorax in dogs requires further study (14).

In conclusion, our results show the site assessed (CDB vs. CTS) for lung sliding and the sonographic signs (AACS, lung sliding) evaluated with different POCUS protocols can influence the accuracy of diagnosing pneumothorax in PPV canine cadavers placed in sternal recumbency. However, these findings should be interpreted with caution as they may not be applicable to spontaneously breathing live patients. Prospective clinical studies with larger volume pneumothorax and reference standard CT are required to further define the accuracy of sonographic findings and the ideal sites to assess in dogs with pneumothorax.

## Data Availability

The raw data supporting the conclusions of this article will be made available by the authors, without undue reservation.

## Supplementary material

Supplementary Table 1 Signalment, baseline pleural and lung ultrasound (POCUS) findings, pneumothorax assignment, and post-study findings for canine cadavers included in the study. PTX, pneumothorax; PPV, positive pressure ventilation.

Supplementary Figure 1 Study flow diagram outlining cadaver screening, exclusion criteria, pneumothorax assignment, blinded pleural and lung ultrasound (POCUS) assessment, and post-study thoracic radiography.
