# Supplementary material for: Comparison of ultrasound probe location and sonographic findings used for the evaluation of pneumothorax in canine cadavers: a pilot study
Source: Front Vet Sci. 2026 May 25;13:1707807. doi: 10.3389/fvets.2026.1707807 (PMC13244080; doi:10.3389/fvets.2026.1707807)
Supplement: Supplementary Table 1 — Signalment, baseline pleural and lung ultrasound (POCUS) findings, pneumothorax assignment, and post-study findings for canine cadavers included in the study. PTX, pneumothorax; PPV, positive pressure ventilation. [file Table_1.docx]

| **Cadaver ID** | **Weight (kg)** | **Signalment**  **Body condition score (/9)** | **Pre-Study POCUS BL Notes**  **PTX = pneumothorax** | **Pneumothorax classification/alteration**  **Left Right** | | **Post-study Scan** |
| --- | --- | --- | --- | --- | --- | --- |
| C1 | 27.7 | Black and white MI collie  5/9 | Partially frozen 01/11/23, submerged in cool water.  **Pre scan:**  L: Scant PTX  R: No PTX | Added 2 ml/kg air - PTX | Control | L: Mild PTX  R: No PTX |
| C2 | 30.1 | Female golden retriever  6/9 | Partially frozen 01/11/23, submerged in cool water.  **Pre scan:**  L: No PTX  R: No PTX | Added 3ml/kg air - PTX | Added 3ml/kg air - PTX | L: Scant PTX  R: Scant PTX |
| C3  X | 64 | Grey mastiff MI  5/9 | Extremely large patient, excluded due significant pre-existing PTX (and patient size - difficult to create lung sliding).  L: Moderate PTX (excluded)  R: Mild PTX |  |  |  |
| C4 | 26.4 | Black and grey mix female  7/9 | **Pre scan:**  L: No PTX  R: No PTX | Control | Added 3ml/kg air - PTX | L: No PTX  R: estimated 5ml/kg PTX |
| C5  X | 26.5 | White and black speckled mix  MN | Partially frozen 01/11/23, submerged in cool water.  Insufficiently thawed  **No lung slide on POCUS, excluded from study** |  |  |  |
| C6  X | 25.2 | Cream shepherd husky mix MN  4/9 | Insufficiently thawed 01/11/23; no lung sliding; left overnight at room temperature.  **No lung slide on POCUS, excluded from study** |  |  |  |
| C7 | 18.9 | Black and white collie mix Female  4.5/9 | **Pre scan:**  L: Mild PTX  R: No PTX | PTX | 3ml/kg air - PTX | L: Mild PTX  R: PTX at the border of ⅓ and ⅔ lung |
| C8 | 17.6 | Brindle pitbull MI  4/9 | **Pre scan:**  L: No PTX  R: No PTX | Control | Control | L: No PTX  R: No PTX |
| C9 | 13.5 | Black, white and tan corgi mix Female  4/9 | **Pre scan:**  L: No PTX  R: No PTX | Control | Control | L: No PTX  R: No PTX |
| C10  X | 27.5 | Grey and white husky MN  4/9 | L: Scant effusion, no PTX  R: Mild fluid, no PTX  Insufficiently thawed 01/11/23; no lung sliding; left overnight at room temperature.  **No lung slide on POCUS, excluded from study** |  |  |  |
| C11 | 16.6 | Black and white  Mix Female  4/9 | **Pre scan:**  L: Mild PTX  R: No PTX | PTX | Control | L: Scant PTX  R: No PTX |
| C12 | 22.1 | Black Lab Mix Female  6/9 | L: Moderate PTX  R: Moderate PTX  Excluded due significant pre-existing PTX |  |  |  |
| C13 | 27.3 | Dark Tan Chow  Female  5/9 | **Pre scan:**  L: No PTX  R: No PTX | Control | 3ml/kg air - PTX | L: No PTX  R: Mild PTX |
