# Supplementary figures and images for: Comparison of ultrasound probe location and sonographic findings used for the evaluation of pneumothorax in canine cadavers: a pilot study
Source: Front Vet Sci. 2026 May 25;13:1707807. doi: 10.3389/fvets.2026.1707807 (PMC13244080; doi:10.3389/fvets.2026.1707807)

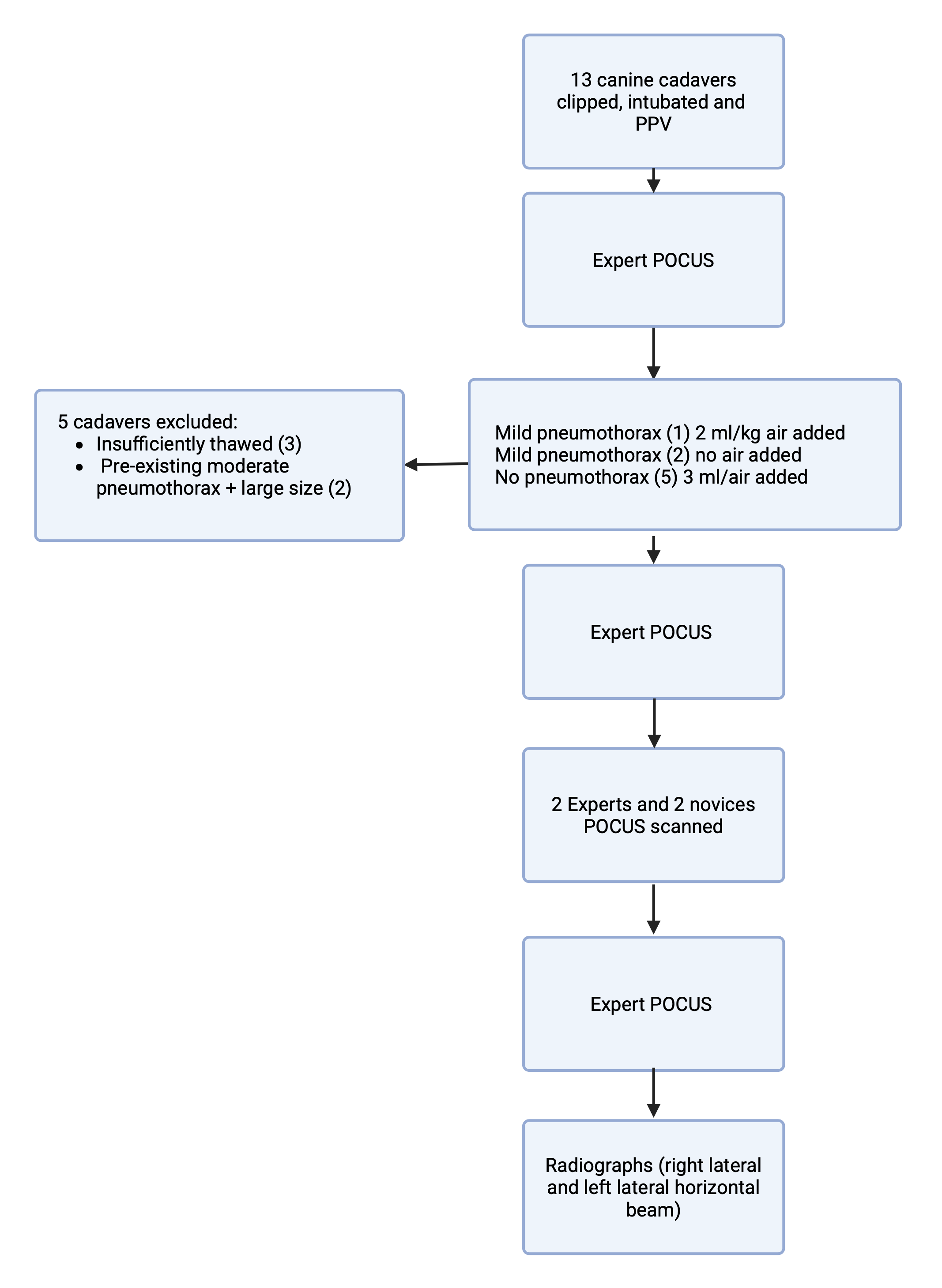

Supplement: Supplementary Figure 1 — Study flow diagram outlining cadaver screening, exclusion criteria, pneumothorax assignment, blinded pleural and lung ultrasound (POCUS) assessment, and post-study thoracic radiography. [file Image_1.png]
